# The role of potassium in depth profiling of the tumor border in bone-invasive oral cancer using laser-induced breakdown spectroscopy (LIBS): a pilot study

**DOI:** 10.1007/s00432-023-05411-9

**Published:** 2023-09-16

**Authors:** Philipp Winnand, K. Olaf Boernsen, Mark Ooms, Marius Heitzer, Matthias Lammert, Jörg Eschweiler, Frank Hölzle, Ali Modabber

**Affiliations:** 1 Department of Oral and Maxillofacial Surgery, University Hospital RWTH Aachen, Pauwelsstraße 30, 52074 Aachen, Germany; 2 Advanced Osteotomy Tools AG, Wallstrasse 6, 4051 Basel, Switzerland; 3 Institute of Pathology, University Hospital RWTH Aachen, Pauwelsstraße 30, 52074 Aachen, Germany; 4 Department of Orthopaedics, Trauma and Reconstructive Surgery, University Hospital RWTH Aachen, Pauwelsstraße 30, 52074 Aachen, Germany

**Keywords:** Bone invasion, Depth profiling, Head and neck cancer, Laser-induced breakdown spectroscopy (LIBS), Microscopic tumor spread, Potassium

## Abstract

**Purpose:**

Microscopic tumor spread beyond the macroscopically visible tumor mass in bone represents a major risk in surgical oncology, where the spatial complexity of bony resection margins cannot be countered with rapid bone analysis techniques. Laser-induced breakdown spectroscopy (LIBS) has recently been introduced as a promising option for rapid bone analysis. The present study aimed to use LIBS-based depth profiling based on electrolyte disturbance tracking to evaluate the detection of microscopic tumor spread in bone.

**Methods:**

After en bloc resection, the tumor-infiltrated mandible section of a patient’s segmental mandibulectomy specimen was natively investigated using LIBS. Spectral and electrolytic depth profiles were analyzed across 30 laser shots per laser spot position in healthy bone and at the tumor border. For the histological validation of the lasered positions, the mandibular section was marked with a thin separating disc.

**Results:**

Solid calcium (Ca) from hydroxyapatite and soluble Ca from dissolved Ca can be reliably differentiated using LIBS and reflect the natural heterogeneity of healthy bone. Increased potassium (K) emission values in otherwise typically healthy bone spectra are the first spectral signs of tumorous bone invasion. LIBS-based depth profiles at the tumor border region can be used to track tumor-associated changes within the bone with shot accuracy based on the distribution of K.

**Conclusion:**

Depth profiling using LIBS might enable the detection of microscopic tumor spread in bone. In the future, direct electrolyte tracking using LIBS should be applied to other intraoperative challenges in surgical oncology to advance rapid bone analysis by spectroscopic–optical techniques.

**Supplementary Information:**

The online version contains supplementary material available at 10.1007/s00432-023-05411-9.

## Background

Bone-infiltrating tumors with microscopic bone spread constitute a special cancer phenotype, in which minimal and macroscopically invisible changes in bone contrast with fatal survival effects (Singh et al. 2019). Microscopic tumor spread beyond the macroscopically visible tumor mass in bone increases spatial complexities at the bone tumor resection margins and may lead to the loss of local tumor control (Lubek and Magliocca 2017). This problem cannot be countered during surgery due to the general lack of rapid analysis techniques for bone, which must first be decalcified over several days prior to its histological evaluation (Savi et al. 2017). The detection of microscopic tumor spread in bone using tumor depth profiling could be based on tracking electrolyte disturbances, as they are critical contributors to tumor invasion in bone (Del-Río-Ibisate et al. 2021; Xie et al. 2022).

In general, ion channels are crucially involved in the hypoxic and metabolic reprogramming of tumor cells, as defined by the Warburg effect (Iorio et al. 2019). While deregulating cellular metabolism is a hallmark of cancer development and progression (Hanahan and Weinberg 2011; Hanahan 2022), ion channel-dependent cellular functions appear to be critically involved in the transformation from healthy to malignant cells and contribute, in principle, to each hallmark of cancer (Prevarskaya et al. 2018). Specifically, head and neck cancer cells are characterized by a particularly high heterogeneity of molecular alterations. For head and neck cancer, in which ion channel dysregulation mainly affects potassium (K), sodium (Na), and calcium (Ca) (Del-Río-Ibisate et al. 2021), ion channel gene-based prediction models have already been developed for prognosis and therapy guidance (Han et al. 2022).

Advanced stages of head and neck tumors with bone involvement imply an increase in the complexity of therapeutic management with the need for adjuvant therapy (German Cancer Society 2021; National Comprehensive Cancer Network 2022) and a massive worsening of prognosis (Tu et al. 2022). The underlying mechanisms of bone invasion can be hypothetically explained at the cellular level. Contrary to earlier considerations, tumorous infiltration of the bone does not occur via direct destruction of the bone by the tumor cells. Instead, oral cancer cells indirectly activate osteoclasts, which dissolve solid bone hydroxyapatite. This leads to local or distant osteolytic lesions, which provide space for tumor growth along the bone (Maurizi et al. 2021; Vaassen et al. 2017). Because these theoretical considerations cannot currently be proven using a direct detection method of electrolyte changes within bone-infiltrating tumor tissue, the hypothetical potential of direct electrolyte tracking cannot yet be applied to practical intraoperative challenges in surgical oncology.

Laser-induced breakdown spectroscopy (LIBS) is a well-known method of analyzing the surfaces of solid materials. In the past, the application of LIBS to biological tissue yielded limited results because the reproducibility of individual spectra was often poor (Moros and Laserna 2019). Recently, we proposed an experimental LIBS setup and a robust evaluation method of LIBS spectra that were optimized for the reproducible evaluation of natively lasered human biomaterial (Winnand et al. 2022). On this basis, we trained tissue detection algorithms on healthy bone and bone-infiltrating tumor tissue from 15 different patients with LIBS, where K could be identified as a crucial discriminator (Winnand et al. 2023). In this previous study, the tumor border region was deliberately excluded to avoid compromising tissue purity.

As a continuation of our previous work, the present article focuses on the LIBS spectra themselves in the tumor border region. By directly detecting electrolyte processes, spectra from the tumor border regions are used to evaluate LIBS-based depth profiles, thereby identifying microscopic tumor spread in bone. In addition, the LIBS spectra are used to provide further evidence of the complex mechanisms of bone invasion processes by oral cancer cells. For a comprehensive understanding of LIBS on human tissue samples, both the biological and the pure spectroscopic views are discussed in this paper.

## Methods

### Handling protocol

After en bloc resection, a segmental mandibulectomy specimen with bone invasion was obtained. The main specimen was macroscopically assessed and sawed in the native state into cross sections, each with a thickness of 4 mm. Together with the pathologist, cross sections were selected that showed clear tumor invasion into the bone and were not needed for routine histology. These selected cross sections were natively frozen and stored at − 20 °C until the performance of the LIBS experiments. Prior to the analysis, the samples were cleaned with 0.9% NaCl and dried roughly with sterile compresses. The wet samples were placed on ash-free round filters (Binzer and Munktell Filter, Battenberg, Germany) for 1 h to reduce the water content and absorb fluid from the intercellular space.

### Experimental setup and LIBS experiments

The LIBS experiments were performed using the AOT LIBS system (Advanced Osteotomy Tools, Basel, Switzerland) with a Q-switched Nd:YAG laser (frequency = 10 Hz; *λ* = 1064 nm) and a spectrometer range between 200 and 870 nm. The experimental setup has been described in previous publications in detail (Winnand et al. 2022, 2023). In the natively lasered mandible section with tumorous bone infiltration, the following elements and species were detected: barium, Ca, calcium hydroxide (CaOH), calcium oxide (CaO), carbon, chromium, cobalt, cyanide, hydrogen (H), K, magnesium (Mg), Na, nitrogen (N), oxygen (O), strontium, and titanium.

**Fig. 1:**
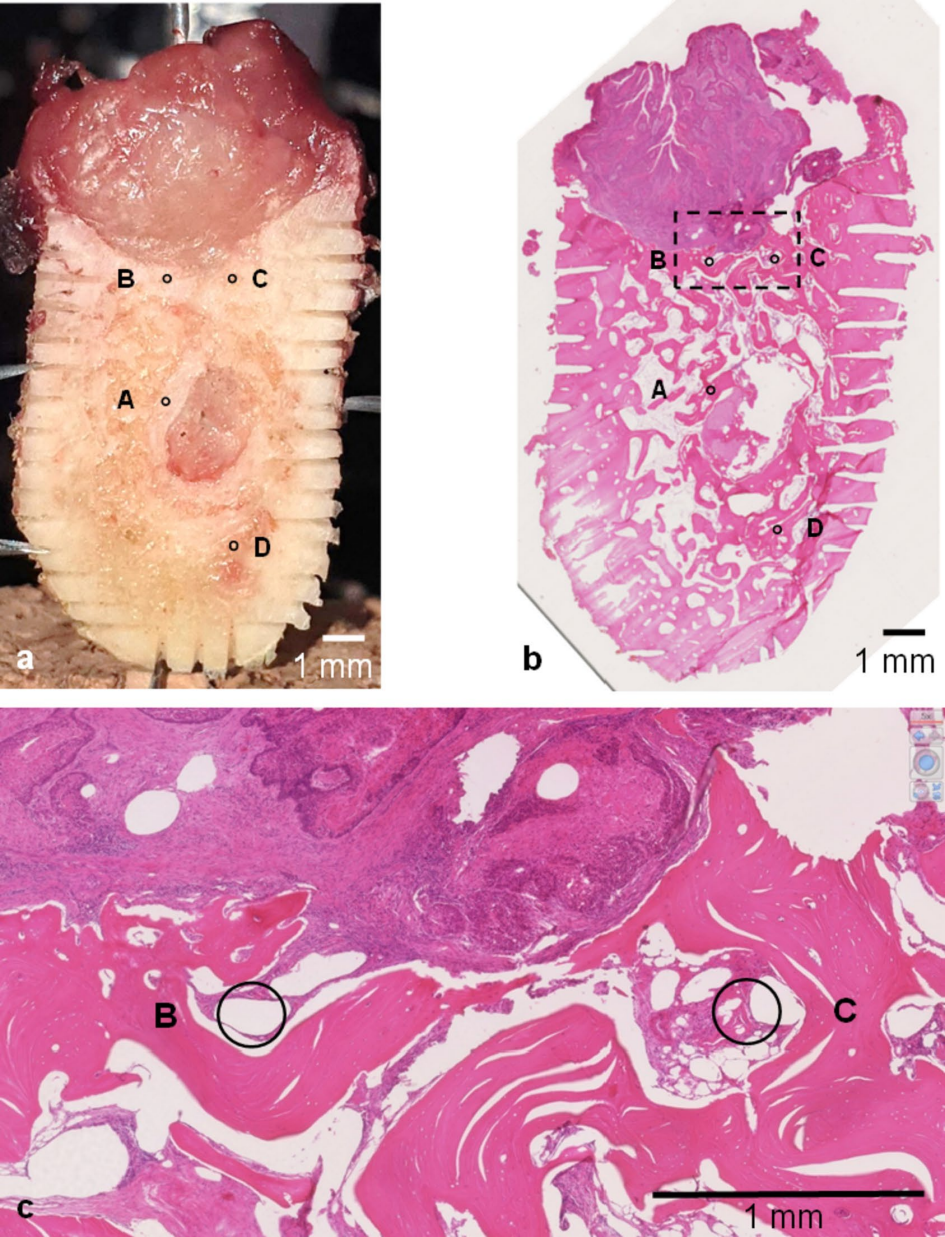
Mandible section with significant tumorous bone invasion. Bone section from a segmental mandibulectomy specimen with vertical and horizontal markings to histologically validate the laser spot positions A, B, C, and D in the H&E stained section. **a** Macroscopic view. **b** Microscopic (0.5× magnified) view with the corresponding positions. **c** Microscopic (5× magnified) view with a focus on spots B and C, which were acquired in the outgrowths of tumor-associated stromal tissue in the bone–tumor interface

LIBS was always performed with 30 laser shots at defined spots in healthy bone substance and at the tumor border region (Fig. 1). The manual LIBS-based examination of one mandible section took 30 min. Markings placed on the mandibular section with a thin cutting disc secured the position of the mandible section for the histological validation of the lasered positions. For the histological validation of the lasered positions by a dedicated pathologist, the mandibular bone section was fixed in paraformaldehyde (10%), decalcified in EDTA (10%) for 14 days, paraffin-embedded, sectioned, and stained in H&E following the LIBS experiments.

For a better understanding of the observed LIBS spectra on natively lasered human biomaterial, water-free calcite (CaCO_3_) was also analyzed using LIBS (Fig. S1).

### Technical considerations and physical dimensions of LIBS microbiopsies

Single-shot sensitivity is a prerequisite for generating a depth profile of a specific laser position in the tissue. Technically, the new LIBS system (Advanced Osteotomy Tools AG, Basel, Switzerland) provides a full emission spectrum from every laser shot. The penetration depth of a single shot depends, in particular, on the material to be lasered and is approximately 3.27 μm as measured in dried cortical bone. Thus, with a laser spot diameter of 240 μm, a cylindrical volume of 0.0081 mL is completely evaporated and heated to several thousand degrees. During the plasma desorption process, which lasts several nanoseconds, the conditions of the cell ensemble in the lasered cylindrical microsphere can no longer be changed (Lei 2012). Therefore, the electrolyte conditions within the microsphere can be measured directly from the breakdown processes and the excitation of atoms and molecules with each laser shot. In this way, 30 individual LIBS emission spectra can be used for a depth profile of approximately 100 μm to assess the tissue heterogeneity of bone substance on a layer-by-layer basis.

It should be noted that the signal intensities and peak areas of the different elements should not be compared against each other, since each element has its own light emission behavior and calibration curve. The element-specific sensitivity of LIBS can be roughly estimated using a mathematical simulation of the LIBS process by the Saha/LTE (local thermodynamic equilibrium) equation (Kramida et al. 2020) for a mixture of highly abundant elements. With LIBS, Ca can be detected at a sensitivity of 14 times that of K. Accordingly, in LIBS spectra with equal peak areas of Ca and K, the measured microsphere actually contains roughly 14 times more K than Ca. It should also be noted that more than 50 different emission lines are observed for Ca in the LIBS spectra, not just two main emission lines (as is the case for Na, K, and Mg).

### Spectra processing

The LIBS spectra were collected without any averaging. Normalization of the data is crucial for further statistical analysis (Guezenoc et al. 2019). For tissue data, the very limited and constantly changing number of peaks within the LIBS spectra and the very high-intensity dynamics of the recorded spectra pose particular challenges for any kind of normalization.

Maximal intensity normalization (also known as base peak intensity normalization) works independently of the number of measured emission peaks and has been successfully applied to LIBS data previously by Singh et al. (2016). For our data, this kind of normalization best corrects for the water-quenching effect of wet tissue samples.

First, the spectrum baseline was carefully subtracted, followed by maximal intensity normalization for each spectrum. The LIBS measurements on native cross sections of the segmental mandibulectomy specimens resulted in 71 emission lines, which were used for further analysis.

However, LIBS spectra suffer from the Stark effect, which leads to the simultaneous broadening of the peaks and a decrease in intensity (Messaoud Aberkane et al. 2020). No excitation photons are lost in this process, so the peak area best covers this effect. This type of correction for calibration curves of reference spectra has been shown previously (Winnand et al. 2022). To apply this strategy to real-life samples, a bin of a defined width was manually specified for each possible emission peak. This kind of binning (also known as bucketing) is commonly used in the quantification of nuclear magnetic resonance data (Emwas et al. 2018). The calculation of the individual peak areas was based on these defined bins. In this way, a set of 71 peak area or bin values was extracted from each individual spectrum, as previously described (Winnand et al. 2022, 2023).

### Quality control

The LIBS process is fundamentally dependent on several factors that cannot be controlled (Winnand et al. 2022) and therefore require special attention when working with natively lasered human samples. In general, the signal intensities of LIBS spectra can be compromised by tissue heterogeneity, moisture-induced signal quenching, the low concentrations of metal atoms and molecules, and low plasma temperature evolution during the absorption of the analyzed material (Singh and Thakur 2007; Sanghapi et al. 2018; Winnand et al. 2022). Specifically, this study needed to consider the different tissue properties of solid bone as well as tumorous and non-tumorous tissue cells to properly assess the biological significance of the LIBS spectra.

A quality control (QC) step was performed to compare only the spectra that conformed to simple QC rules and were measured within a well-defined range under identical conditions. Spectra below a certain threshold and those with any kind of peak saturation were excluded. Reactions between the high laser beam flux and dust particles can produce airborne spectra above the tissue surface, which can be detected by the eye as bright plasma flashes in the air in front of the tissue. Because these LIBS spectra are dominated by O and N emission lines (Fig. S2), they are completely different from real tissue spectra and were, therefore, discarded. Ultimately, defined QC criteria compensate for the limitations that occur even with the best normalization methods. For this patient sample, the aforementioned QC criteria resulted in the exclusion of approximately 5% of the collected spectra. Due to the high number of measured LIBS spectra and the possibility of interpolation, this small loss of information was acceptable.

The correct assignment of elements and wavelengths was first ensured by comparison with spectra recorded in a previous publication on reference solutions containing pure CaCl_2_ and KCl_2_ (Winnand et al. 2022), and subsequently by spectra simulation of the individual elements or their mixtures by the Saha/LTE simulation (Safi et al. 2019) using the National Institute of Standards and Technology database (Kramida et al. 2020). Microsoft Excel was used to process and analyze the spectra.

## Results

### Spectral depth profiling in healthy bone material

**Fig. 2:**
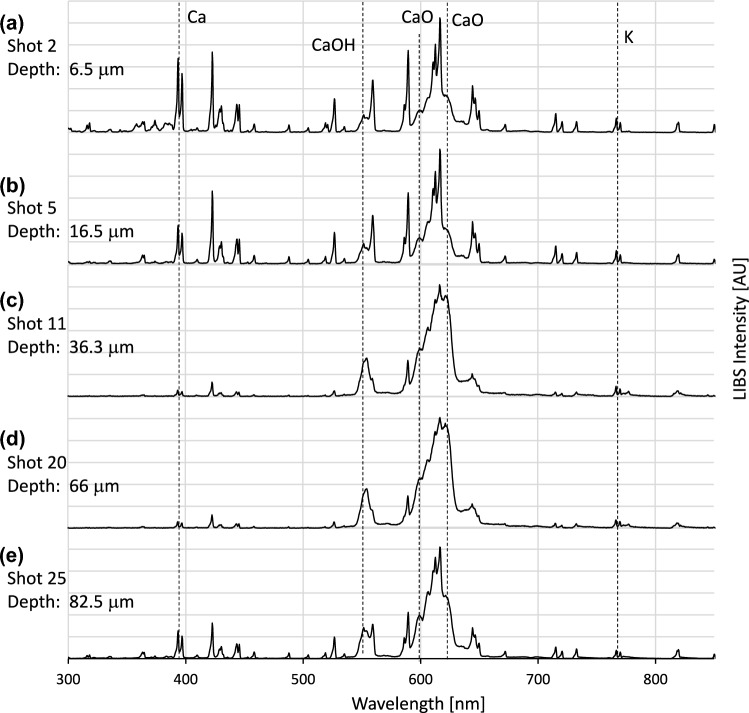
Spectral depth profiling in healthy bone material. Typical LIBS emission spectra of healthy bone at laser spot position A. Selected depth profile spectra at laser shot 2 (**a**), shot 5 (**b**), shot 11 (**c**), shot 20 (**d**), and shot 25 (**e**). Note the consistently low K emission lines in the spectra from healthy bone. All spectra have been normalized to base peak intensity (BPI)

Figure 2 shows the typical emission spectra (base peak intensity normalized) out of 30 laser shots from a given laser position (spot A) in healthy bone. Three typical kinds of LIBS emission spectra were observed in the healthy bone substance.

The first two spectra (Fig. 2a and b) displayed specific and narrow calcium emission lines (393.5 nm, 397.3 nm, and 422.6 nm) and moderate CaO and CaOH molecule emission bands (545–595 nm and 607–615 nm). Dry (water-free) CaCO_3_ showed similar emission spectra but had much smaller CaO and CaOH bands. A film of water or high humidity did not change the spectrum (Fig. S1).

In contrast, the emission spectra of the next two shots lacked sharp solid Ca lines but were characterized exclusively by intense CaO and CaOH bands (Fig. 2c and d). These spectra were comparable to those of wet soluble Ca, which showed very intense CaO and CaOH emission bands without other Ca emission lines (Fig. S3).

The last shot covered a depth of approximately 83 μm and showed a combination of the aforementioned spectra, with few narrow Ca lines and intense CaO and CaOH bands (Fig. 2e).

After 30 laser shots, no loss of intensity was observed as long as the cell types and the microstructure at the respective lasered position did not change. After data processing, the sum of all bins was nearly constant (Fig. S4).

### Reproducibility and depth profiling of different elements in healthy bone substance

**Fig. 3:**
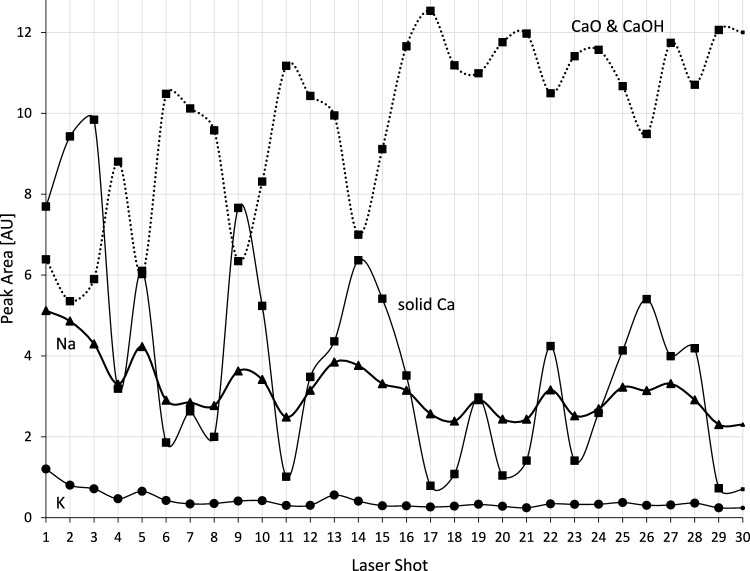
Depth profiling of different elements in a healthy bone substance. Depth profiles of different elements in healthy bone (laser spot position A). The contents of K, Na, solid Ca, and soluble Ca are shown by their emission lines, covering approximately 100 μm. The strong changes in the solid Ca and soluble Ca emission lines reflect the heterogeneity of the tissue itself. Dotted line with squares: Soluble Ca, measured as CaO and CaOH species. Thin solid line with squares: Solid Ca from hydroxyapatite. Bold line with triangles: Na. Bold line with round dots: K. The missing dots at laser shot 30 indicate LIBS spectra that did not fulfill the QC criteria

To confirm the accuracy of the single LIBS measurements, pure, healthy bone was investigated over the given range (Fig. 3). The emission line spectra of the healthy bone substance were characterized by strong changes in solid Ca and soluble Ca (CaO and CaOH species). In a regular manner, increases in solid Ca emission lines were accompanied by decreases in soluble Ca emission lines.

Over the complete range of 30 shots, the healthy bone showed constantly low levels of K and Na, with minor shot-to-shot fluctuations against the strong changes in the tissue heterogeneity.

### Depth profiling on the tumor border

**Fig. 4:**
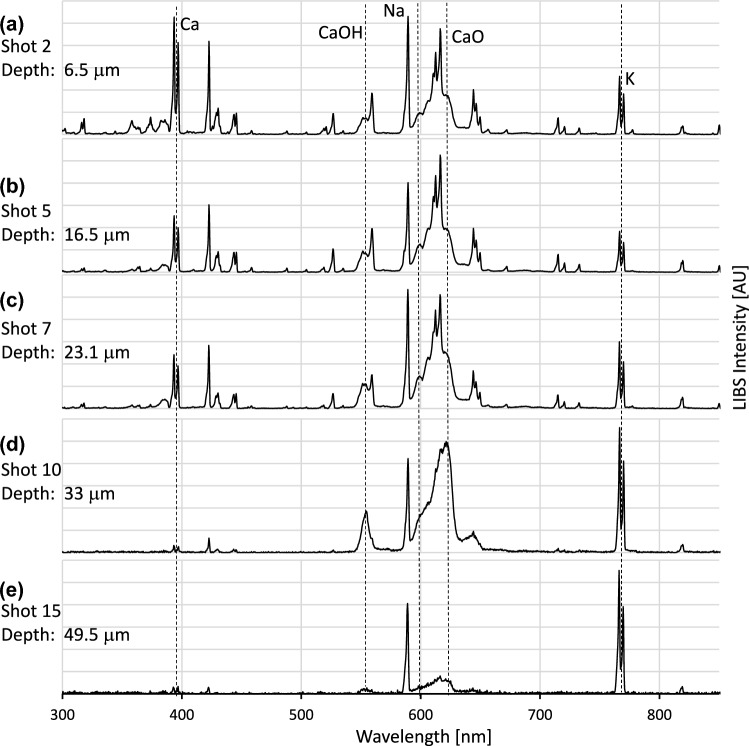
Spectral depth profiling on the tumor border. Typical LIBS spectra of tumor border area, measured at laser spot position B. Selected depth profile spectra at laser shot 2 (**a**), shot 5 (**b**), shot 7 (**c**), shot 10 (**d**), and shot 15 (**e**). Note the increase in K with the increasing depth of laser shots

Figure 4 represents five spectra from a spot that appeared macroscopically healthy and was directly located at the tumor border. The first three emission spectra revealed signal intensities of Ca similar to those in the spectra of healthy spongiosa (Fig. 2). With increasing depth, the emission of solid Ca decreased, while the peaks of soluble Ca species broadened and the K signal intensities at 766.6 nm and 770.1 nm increased (Fig. 4a, b, and c). As illustrated in Fig. 4d and e, the spectra were dominated by the strong emission of K, with the complete elimination of solid and soluble Ca emission lines.

The histological validation of the tissue below the last laser shot revealed outgrowths of tumor-associated stromal tissue at the bone–tumor interface (Fig. 1).

### Depth profiling by selected electrolytes near the tumor border

**Fig. 5:**
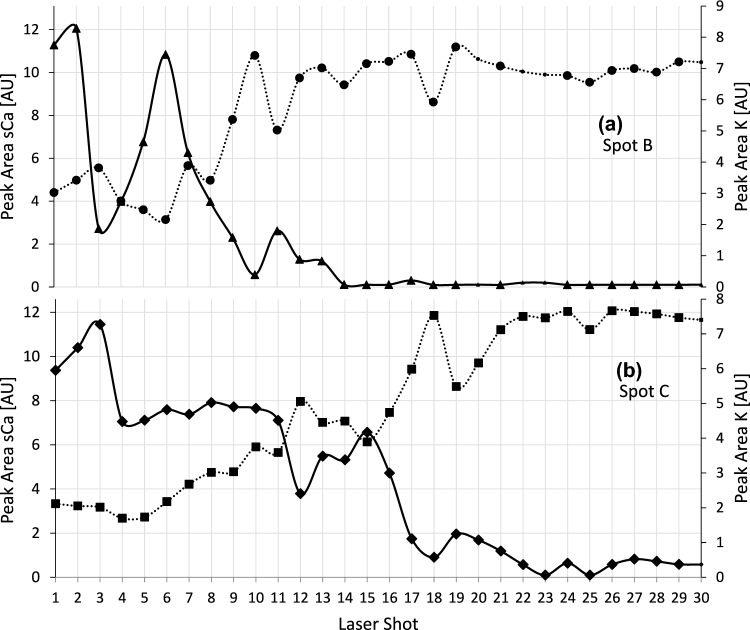
Depth profiling by selected electrolytes near the tumor border. Depth profiles of K and solid Ca at two positions (laser spot position B [a] and laser spot position C [b]) from the tumor border. The figures show a steady increase in K and a decrease in solid Ca with the increasing depth of shots. The tumor-associated stromal tissue starts at laser shot 10 (**a**) and laser shot 17 (**b**). The tumor-associated stromal tissue in both spots reached similar peak areas for K. Solid lines used for solid Ca. Dotted lines were used for K. Missing dots: Spectra that did not fulfill the QC criteria and were discarded. The vertical scale on the left represents the peak area of solid Ca, and the vertical scale on the right represents the peak area of K

To demonstrate the accuracy and reproducibility of the LIBS measurements, the K levels were observed over the complete range of 30 shots at two positions spaced 2 mm apart. Figure 5 shows how the K and solid Ca levels changed as the laser “drilled” into the tissue at the tumor border.

The two positions showed very similar patterns with respect to the peak areas of K and solid Ca. In both positions, solid Ca was high on the macroscopically healthy surface. After 10 (laser spot position B) and 17 (laser spot position C) laser shots, increases in K and decreases in solid Ca were observed. In the deepest layers, the peak areas of K clearly dominated over the peak areas of solid Ca. Both positions were histologically validated as tumor-associated stromal tissue at the bone–tumor interface (Fig. 1).

## Discussion

Microscopic tumor spread beyond the macroscopically visible tumor mass in bone is observed in 10% of cases, with a mean microscopic bone tumor spread of 0.71 cm (Singh et al. 2019). The detection of microscopic tumor spread in bone must, therefore, be a key factor in the oncological safety of new tumor detection techniques such as LIBS, which must reliably detect pathological processes but also reliably exclude them in healthy bone. After demonstrating the differentiation between clearly healthy and tumorous tissue using LIBS-based diagnostics (Winnand et al. 2023), spatial complexities at the tumor border can now be reflected by depth profiles, for which single-shot sensitivity and the highly sensitive detection of different states in healthy bone are crucial prerequisites.

The spectral and electrolytic depth profiles in healthy bone (Figs. 2 and 3) confirm the first trends of the spectra in healthy bone substance, which have been shown previously (Winnand et al. 2023). Due to the high Ca content of mineralized healthy bone (Eagle et al. 2015), different states of healthy bone substance can be characterized by the emission spectra of Ca. Solid Ca, corresponding to the hydroxyapatite of the bone, appears to be reliably detectable with LIBS (Fig. 2a and b; Fig. S1). Considering the elementary crystal cell of hydroxyapatite, as given by the formula Ca_5_(OH)_2_(PO4)_4_, the existence of two OH groups on five Ca atoms might be the reason for the detection of soluble Ca species (CaO and CaOH) in the LIBS spectra of healthy bone. The intense CaO and CaOH species may not only originate from the incomplete breakdown of the crystal structure of hydroxyapatite but also indicate states where solid Ca is dissolved (Fig. 2c and d; Fig. S3).

The reliable LIBS-based differentiation of solid Ca from hydroxyapatite and soluble Ca from dissolved Ca, which both most closely reflect the natural heterogeneity of healthy bone material (Pernelle et al. 2017), must be highlighted as the first key finding of the current study. As an extension of research by others, who have previously analyzed single spectra in the bovine mandible (Mehari et al. 2014; Rohde et al. 2017), and as a continuation of our previous work (Winnand et al. 2023), this paper demonstrates that depth profiles of approximately 100 μm can be reproducibly taken in bone without any loss of LIBS signal intensity (Fig. S4). The consistently low level of both K lines at 766.6 nm and 770.0 nm in all LIBS emission spectra from healthy bone represents the second key finding of this study, supporting our previously formulated hypothesis that the absence of K is a strong indicator of healthy bone (Winnand et al. 2023).

These two key findings are essential for the interpretation of depth profiles at the tumor border, where bone invasion is mediated by fibrous stroma tissue at the interface of resorbing bone and bone-invading tumor cells (Elmusrati et al. 2017; Ishikuro et al. 2008; Shan et al. 2022a, b). The superficial spectra at the tumor border were similar in principle to the healthy bone spectra (Fig. 2). However, the broadening of the CaO and CaOH bands was accompanied by a strong increase in K emission lines (Fig. 4a, b, and c). The simultaneous detection of K and soluble Ca is not typical of healthy bone spectra and may indicate the tumor-associated dissolution of solid Ca from bone hydroxyapatite. The direct LIBS-based measurement of soluble Ca species at the tumor border of bone-infiltrating oral cancer might visualize processes of osteoclastogenesis, which are key steps in oral cancer bone invasion (Maurizi et al. 2021), and might now provide unequivocal evidence for the orchestrating role of Ca in the initiation of bone-invasion mechanisms (Lee et al. 2016; Xie et al. 2022).

The deep spectra at the same laser spot position, characterized by highly dominant K emission lines (Fig. 4d and e), then, corresponded with tumor-associated stromal tissue, which was histologically validated at the tumor–bone interface (Fig. 1). The detection of tumor-related changes in bone substance and tumorous stroma within a depth profile of 100 μm at the tumor border confirms the direct link between tumor-associated processes of osteoclastogenesis and tumor stroma, as previously suspected by others (Elmusrati et al. 2017; Ishikuro et al. 2008). As a significant extension to our previous LIBS study, in which the tissue detection algorithms were developed excluding the tumor border (Winnand et al. 2023), the bone–tumor interface can now be reliably assessed using LIBS-based depth profiles (Fig. 5).

The results of this study must be evaluated in the context of minor limitations. The sample stage was moved manually, which meant aiming the laser spot positions by eye. Because of the tissue-ablating nature of the LIBS process, not every laser shot within a 100 μm depth profile, but the most superficial layer beyond the LIBS ablation crater, was histologically validated by a pathologist. As discussed in our previous publications, the humidity of biomaterial or tumor samples can be a practical challenge for LIBS, which can be addressed by peak area calculation and normalization of spectra (Winnand et al. 2022, 2023). Additionally, this paper was deliberately limited to representative spectra from a total of 1710 LIBS spectra from a single patient to highlight the underlying principles of tumor invasion into bone and demonstrate the feasibility of LIBS for detecting microscopic tumor spread in bone.

Despite increasing research interest in the tumor–bone interface, studies to date have been mostly limited to immunolabeling (Shan et al. 2022a; Elmusrati et al. 2017), which is blind to the detection of direct electrolyte changes within healthy and tumorous cells. Due to the reproducible applicability of LIBS to human biomaterials (Winnand et al. 2022), direct measurements of electrolyte changes in natively analyzed biomaterial can now be performed and applied to intraoperative challenges in surgical oncology. As demonstrated in a proof-of-principle study on 15 patients with bone-invasive oral cancer, LIBS could be a promising technique for rapid bone analysis and could fundamentally improve intraoperative management (Winnand et al. 2023). The evaluation of LIBS-based depth profiles using electrolyte tracking might enable the real-time detection of macroscopically invisible changes in bone and provide shot-accurate imaging of the bone–tumor interface. Consequently, the use of LIBS depth profiles could reduce spatial complexities at irregular bone tumor resection margins that do not grow strictly vertically into the bone. Furthermore, the LIBS-based detection of microscopic tumor spread in bone could identify high-risk patients in whom the resection margins in bone must then be extended intraoperatively to improve local tumor control. A risk-adapted approach could ensure oncologic safety without generally requiring a resection margin of 15 mm in bone, as required in retrospective studies owing to concerns about microscopic tumor spread (Singh et al. 2019).

Finally, analogous to the differentiation of tumorous tissue and healthy bone (Winnand et al. 2023), K also plays a crucial role in the interpretation of LIBS-based tumor depth profiles. Specifically, increased K values in otherwise typically healthy bone spectra correspond to the first signs of early changes attributable to tumor-related bone invasion. Furthermore, the transition of tumor-associated bone changes into tumorous stroma can be accurately tracked by the distribution of directly measured K at individual positions.

In general, this observation is consistent with the Warburg effect (Iorio et al. 2019) and is in agreement with the literature, in which increased K and Na concentrations have been postulated within living tumor cells (Leslie et al. 2019; Eil et al. 2016; Jessica et al. 2021). In the specific context of head and neck cancer cells, the direct detection of K within the stroma of bone-infiltrating tumor cells now substantiates the promoting effect of K on head and neck cancer cell progression, proliferation, and invasiveness in advanced tumor stages (Del-Río-Ibisate et al. 2021).

Apart from the field of surgical oncology, the knowledge of electrolyte disturbances within living tumor cells could contribute to the further development of targeted therapies to affect K (Chimote et al. 2018, 2020; Gawali et al. 2021; Chandy and Norton 2016) and Na (Zhang et al. 2019) channels, but this must be critically weighed against adverse and immunosuppressive effects (Ganser et al. 2021).

## Conclusion

LIBS allows, for the first time, the direct measurement of the main housekeeping electrolytes of a given cell ensemble in the form of a native microbiopsy. Based on previously trained tissue recognition algorithms and supported by the type of data processing presented here, new insights into the electrolyte distribution in the tumor border area of bone-infiltrating tumors can be gained by the evaluation of tumor depth profiles. The detection of microscopic tumor spread in bone with micrometer-scale depth profiles represents the next step in establishing a LIBS-based rapid bone analysis technique. This technique should be further developed to address additional concrete, practical challenges in oncologic surgery.

## Supplementary Information

Below is the link to the electronic supplementary material.

Supplementary file1 (PDF 279 KB)

## Data Availability

All data generated or analyzed during this study are included in this published article and its supplementary information files.

## Abbreviations

BPI: Base peak intensity
EDTA: Ethylenediamine tetraacetic acid
H&E: Hematoxylin and eosin
LIBS: Laser-induced breakdown spectroscopy
LTE: Local thermodynamic equilibrium
Nd:YAG: Neodymium-doped yttrium aluminum garnet
NMR: Nuclear magnetic resonance
QC: Quality control
