# Supplementary material for: The role of potassium in depth profiling of the tumor border in bone-invasive oral cancer using laser-induced breakdown spectroscopy (LIBS): a pilot study
Source: J Cancer Res Clin Oncol. 2023 Sep 16;149(18):16635–45. doi: 10.1007/s00432-023-05411-9 (PMC10645631; doi:10.1007/s00432-023-05411-9)
Supplement: Supplementary file 1 — Supplementary file1 (PDF 279 KB) [file 432_2023_5411_MOESM1_ESM.pdf]

Supplementary Information:

Journal of Cancer Research and Clinical Oncology

Title: The role of potassium in depth profiling of the tumor border in bone-invasive oral cancer using laser-induced breakdown spectroscopy (LIBS): A pilot study

Philipp Winnand[*;1]; K. Olaf Boernsen[2]; Mark Ooms[1]; Marius Heitzer[1]; Matthias Lammert[3]; Jörg Eschweiler[4]; Frank Hölzle[1]; Ali Modabber[1]

1 Department of Oral and Maxillofacial Surgery, University Hospital RWTH Aachen, Pauwelsstraße 30, D-52074, Aachen, Germany.
2 Advanced Osteotomy Tools AG, Wallstrasse 6, CH-4051 Basel, Switzerland.
3 Institute of Pathology, University Hospital RWTH Aachen, Pauwelsstraße 30, D-52074 Aachen, Germany.
4 Department of Orthopaedics, Trauma, and Reconstructive Surgery, University Hospital RWTH Aachen, Pauwelsstraße 30, D-52074 Aachen, Germany

*Corresponding author:
Dr. med. Philipp Winnand
Department of Oral and Maxillofacial Surgery
University Hospital RWTH Aachen
Pauwelsstraße 30
D-52074 Aachen, Germany
E-Mail: pwinnand@ukaachen.de
ORCID: 0000-0001-8315-7684

Fig. S1 LIBS spectra of calcite and cortical bone compared to LTE simulation

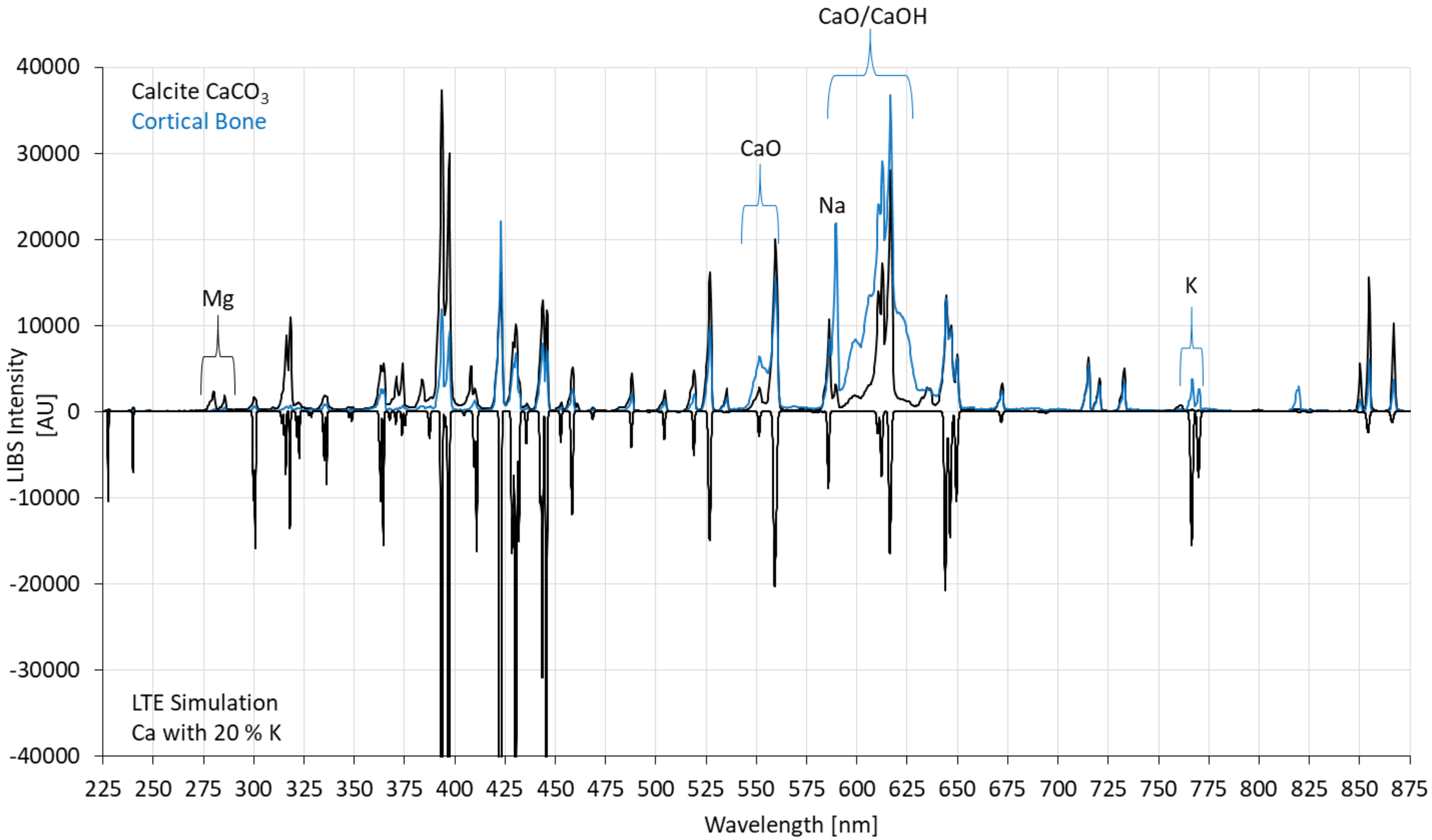

Comparison of LIBS spectra of calcite, cortical bone (laser spot position D, shot 5), and a corresponding LTE simulation (Ca with 20% K). Calcite is a water-free mineral. The corresponding LIBS spectrum shows nearly no CaOH or CaO emission bands, whereas these bands are present in the cortical bone spectrum as OH groups. In the crystal structure of solid bone hydroxyapatite, the Ca ion is associated with six ligands, which are often O atoms. These O and OH ligands could be responsible for the formation of the CaO and CaOH species or result from an incomplete breakdown process within the plasma plume. For a better assignment of the complex emission lines, a Saha/LTE simulation was performed, as shown in the bottom curve. In this way, most of the emission lines could be assigned to their origin elements. Upper curve in black: Calcite ($CaCO_3$). Upper curve in blue: Cortical bone (laser spot position D, laser shot 5). Bottom curve: Saha/LTE simulation of Ca with 20% K.

Fig. S2 LIBS spectrum of an airborne microdroplet

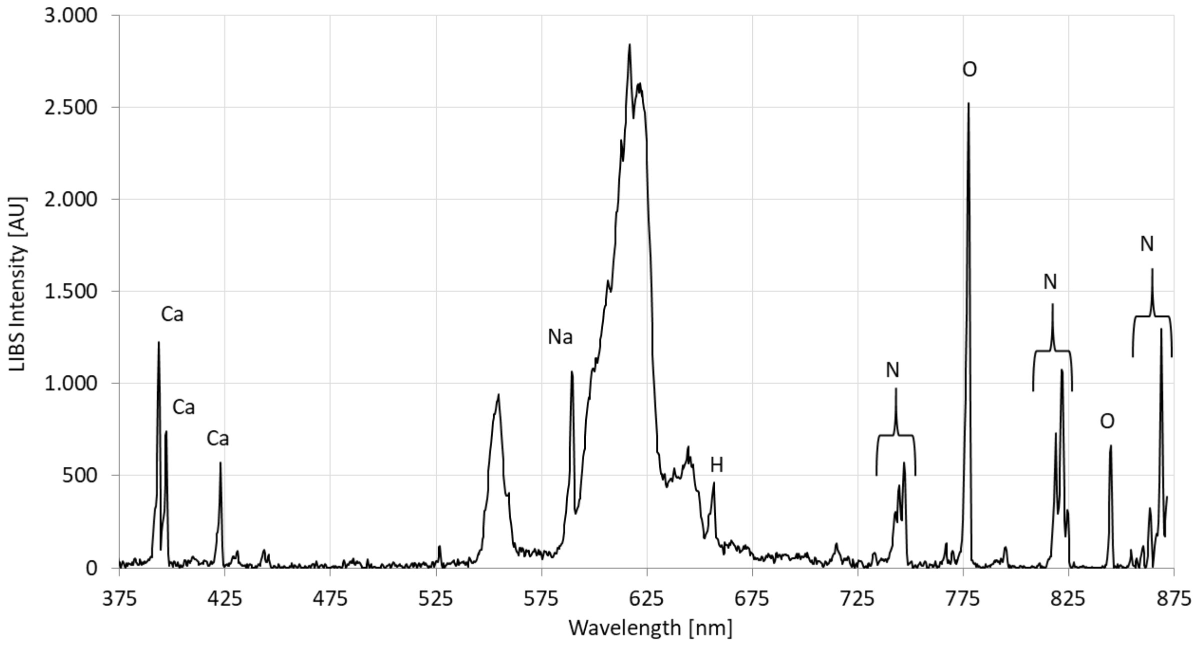

LIBS spectrum of a typical airborne microdroplet from a laser shot desorption process above the tissue surface. This type of laser shot is easily recognized by the small flash of light a few millimeters above the tissue surface. The spectrum itself shows the typical Ca lines and bands but also intense N and O lines. Even the hydrogen beta line became visible in this kind of spectrum. All these new emission lines were generated within the plasma plume by the reaction of water and air. The frequency of this kind of LIBS spectra was low (approximately 1:225). Note that such a spectrum did not meet the QC criteria.

Fig. S3 LIBS spectrum of soluble Ca

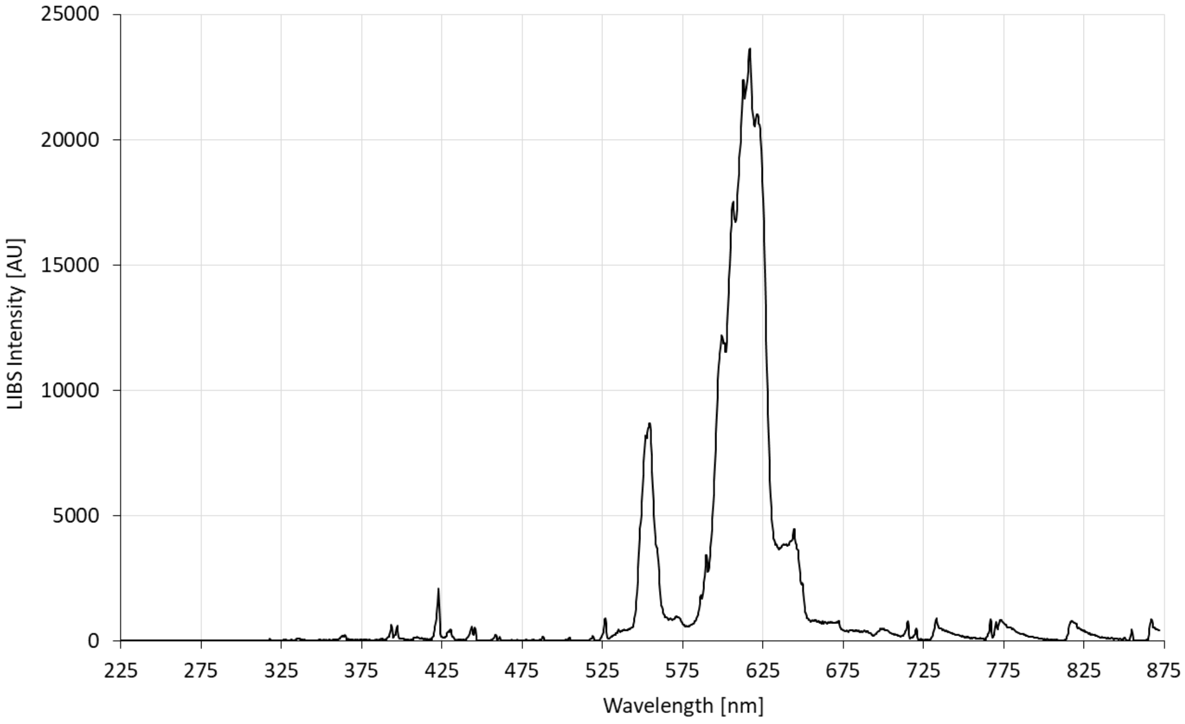

LIBS spectrum of soluble Ca after 60 laser shots, when the sample became dry enough to generate a full LIBS spectrum. The broad emission bands of CaO and CaOH dominate the LIBS spectrum with high intensity, while the solid Ca emission lines are low.

Fig. S4 Reproducibility of LIBS spectra and data processing strategy over 30 shots

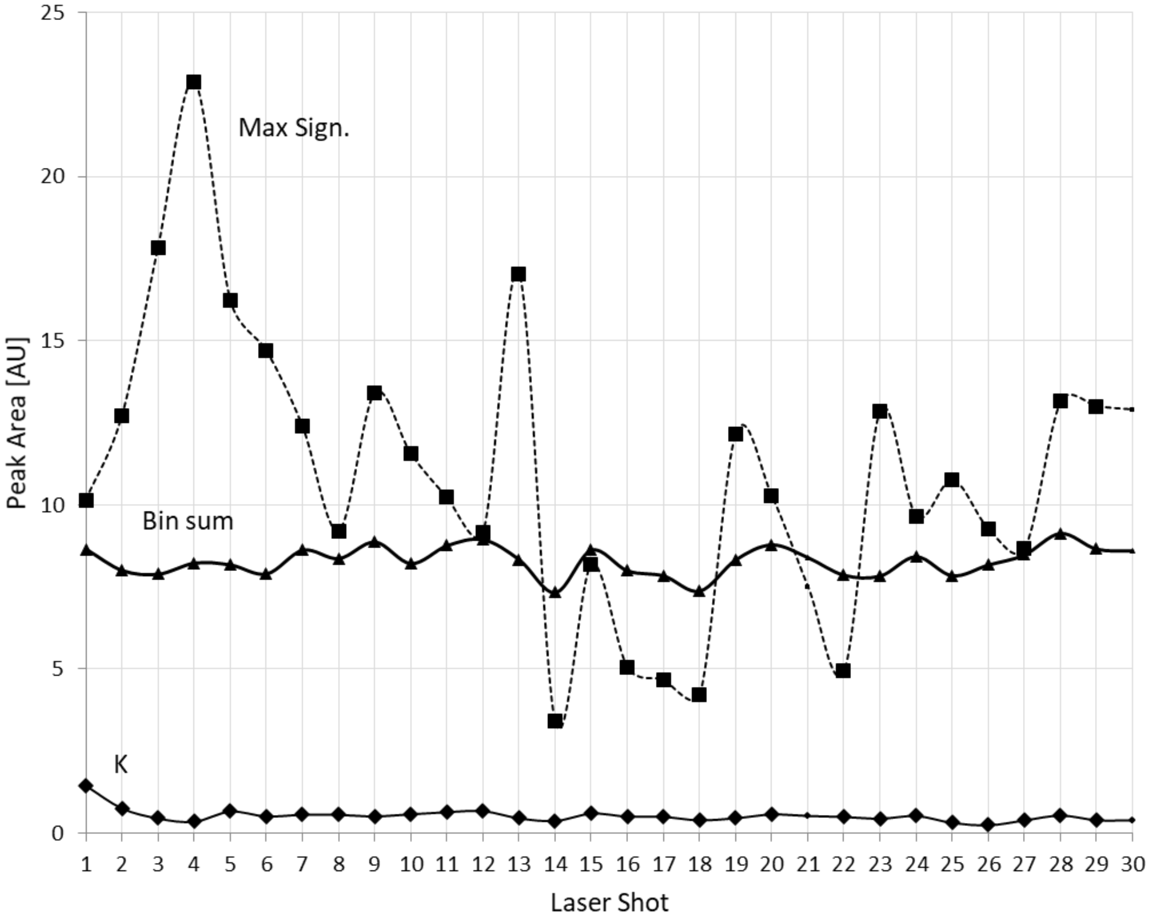

Depth profile of laser spot position D showing the sum of all bins (sum over all calculated peak areas) and the maximum intensity (before normalization) of each emission spectrum. After normalization and binning, the sum of all bin areas is shown as BinSum. For comparison, the peak area of K is shown (vertical scale MaxSig x2000; scale Binsum x10, scale K x1).

This figure shows that there was no loss of signal with the increasing number of laser shots for laser spot position D. It also shows that the chosen data processing strategy resulted in comparable data as shown for BinSum and for K. The emission spectra of laser shots 21 and 30 did not meet all QC criteria and were discarded.
